# Commensal and pathogenic biofilms differently modulate peri‐implant oral mucosa in an organotypic model

**DOI:** 10.1111/cmi.13078

**Published:** 2019-07-17

**Authors:** Alexandra Ingendoh‐Tsakmakidis, Carina Mikolai, Andreas Winkel, Szymon P. Szafrański, Christine S. Falk, Angela Rossi, Heike Walles, Meike Stiesch

**Affiliations:** ^1^ Department of Prosthetic Dentistry and Biomedical Materials Science Hannover Medical School Hannover Germany; ^2^ Institute of Transplant Immunology Hannover Medical School Hannover Germany; ^3^ Translational Center for Regenerative Therapies Fraunhofer Institute of Silicate Research ISC Würzburg Germany; ^4^ Chair of Tissue Engineering and Regenerative Medicine University Hospital of Würzburg Würzburg Germany

**Keywords:** *Aggregatibacter actinomycetemcomitans*, dental implants, host modulation, organotypic oral mucosa, *Streptococcus oralis*

## Abstract

The impact of oral commensal and pathogenic bacteria on peri‐implant mucosa is not well understood, despite the high prevalence of peri‐implant infections. Hence, we investigated responses of the peri‐implant mucosa to *Streptococcus oralis* or *Aggregatibacter actinomycetemcomitans* biofilms using a novel *in* *vitro* peri‐implant mucosa‐biofilm model. Our 3D model combined three components, organotypic oral mucosa, implant material, and oral biofilm, with structural assembly close to native situation. *S. oralis* induced a protective stress response in the peri‐implant mucosa through upregulation of heat shock protein (HSP70) genes. Attenuated inflammatory response was indicated by reduced cytokine levels of interleukin‐6 (IL‐6), interleukin‐8 (CXCL8), and monocyte chemoattractant protein‐1 (CCL2). The inflammatory balance was preserved through increased levels of tumor necrosis factor‐alpha (TNF‐α). *A. actinomycetemcomitans* induced downregulation of genes important for cell survival and host inflammatory response. The reduced cytokine levels of chemokine ligand 1 (CXCL1), CXCL8, and CCL2 also indicated a diminished inflammatory response. The induced immune balance by *S. oralis* may support oral health, whereas the reduced inflammatory response to *A. actinomycetemcomitans* may provide colonisation advantage and facilitate later tissue invasion. The comprehensive characterisation of peri‐implant mucosa‐biofilm interactions using our 3D model can provide new knowledge to improve strategies for prevention and therapy of peri‐implant disease.

## 1 INTRODUCTION

Microorganisms are able to colonize oral surfaces, regardless of whether it is natural, for example, tooth enamel or mucosa, or artificial, for example, titanium implant, and form complex biofilms (G. N. Belibasakis, Charalampakis, Bostanci, & Stadlinger, 2015; Furst, Salvi, Lang, & Persson, 2007; Kolenbrander, Palmer, Periasamy, & Jakubovics, 2010). Different factors (immunodeficiency, systemic disease, environmental factors, and keystone pathogens) can induce a shift in species composition of oral biofilms incorporating more pathogenic bacteria (Graves, Correa, & Silva, 2019; G. Hajishengallis, 2014; G. Hajishengallis & Lamont, 2016). As a result, in people carrying dental implants, peri‐implant diseases might develop (G. N. Belibasakis, 2014; Berglundh et al., 2018). The reversible inflammation of the soft tissue around the implant is termed “peri‐implant mucositis.” The more severe form, which is termed peri‐implantitis, is irreversible and additionally characterised by loss of bone supporting the implant (Berglundh et al., 2018). Moreover, peri‐implant diseases are characterised by high prevalence. A recent meta‐analysis showed that 26% of patients with an implant function ≥5 years develop peri‐implantitis (Dreyer et al., 2018). One reason could be that dental implants are missing Sharpey's fibres and the periodontal ligament leading to a reduced physical barrier of the oral mucosa against bacterial invasion (G. N. Belibasakis, 2014). In order to expand the knowledge about the interaction of the peri‐implant mucosa and oral microbiome, physiologically relevant *in* *vitro* models are required. The *in* *vivo* situation is much better reflected in three dimensional (3D) organotypic models (Antoni, Burckel, Josset, & Noel, 2015). The co‐culture of organotypic oral mucosa models with planktonic bacteria, monospecies biofilms, or even multispecies biofilms facilitated *in* *vitro* studies, which explored the impact of host–microbe interactions (T. Ahlstrand et al., 2017; Andrian, Grenier, & Rouabhia, 2004; Bao, Papadimitropoulos, Akgul, Belibasakis, & Bostanci, 2015; Buskermolen et al., 2016; Diaz et al., 2012; Gursoy, Pollanen, Kononen, & Uitto, 2010; Pinnock, Murdoch, Moharamzadeh, Whawell, & Douglas, 2014). In order to study the soft‐tissue‐implant interface, Chai *et* *al*. developed an organotypic oral mucosa with an integrated implant. However, their model did not include an oral biofilm, which is a key element of the peri‐implant area (Chai et al., 2010; Chai et al., 2013; Chai, Brook, Palmquist, van Noort, & Moharamzadeh, 2012). To the best of our knowledge, an *in* *vitro* model to study the interactions between all three components, implant material, organotypic oral mucosa, and biofilm, is absent.

Balanced immune response maintains the host‐microbe homeostasis and confers oral health. The oral health‐associated symbiotic microbial community consists mainly of gram‐positive *Streptococcus* spp. and *Actinomyces* spp., and dozens of less studied species are present (G. Hajishengallis, 2015; Mombelli, Müller, & Cionca, 2012; Szafranski et al., 2015). The commensal *Streptococcus oralis* belongs to the initial colonizer and is one of the predominant *Streptococcus* spp. in the early biofilm (Diaz et al., 2006) and consequently should have a considerable impact on oral homeostasis. However, little is known about the mechanisms by which *S. oralis* interacts with the host. This knowledge would help to elucidate the role of this microbe in host‐microbiome homeostasis beyond biofilm initiation. The opportunistic pathogen *Aggregatibacter actinomycetemcomitans* is genetically diverse (Kittichotirat, Bumgarner, & Chen, 2016) and can be detected at peri‐implant disease sites (Rams, Degener, & van Winkelhoff, 2014; van Winkelhoff & Wolf, 2000). It expresses various virulence factors and has different strategies to evade host innate defence mechanisms, for example, migration through the epithelium, and binding of different human proinflammatory cytokines (T. Ahlstrand et al., 2017; Dickinson et al., 2011; Herbert, Novince, & Kirkwood, 2016). However, the overall impact including transcriptional response of *A. actinomycetemcomitans* on the oral mucosa remains unclear. Deciphering of how commensal and pathogenic bacteria, that is, *S. oralis* and *A. actinomycetemcomitans*, impact mucosal homeostasis would help to understand peri‐implant pathogenesis and to develop new therapeutic options.

**Figure 1:**
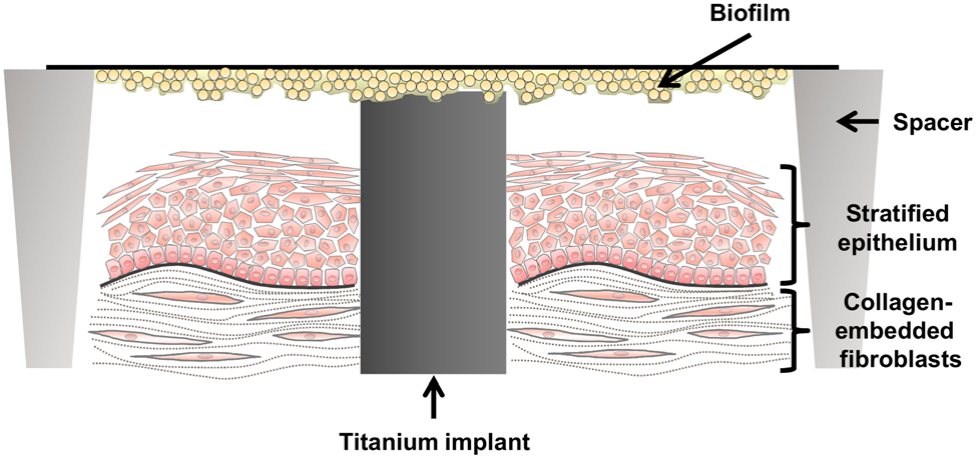
Schematic illustration of the peri‐implant mucosa‐biofilm model. The organotypic oral mucosa with an integrated implant was developed in culture inserts. Spacers with a ring form were placed around the tissue model, which allowed the disposition of the Streptococcus oralis or Aggregatibacter actinomycetemcomitans biofilm on top of the implant. Spacers and implant material have the same height keeping the biofilm planar

The first aim of the present study was to develop an *in* *vitro* peri‐implant mucosa‐biofilm model combining the main three components: the organotypic oral mucosa, an implant material, and an oral biofilm (Figure 1). The second aim was to expand the knowledge about the species‐specific effect of commensals and opportunistic pathogens on the mucosal tissue, by studying the impact of either *S. oralis* or *A. actinomycetemcomitans* biofilms on the peri‐implant mucosa in our unique organotypic model.

## 2 RESULTS

### 2.1 Characterisation of the peri‐implant mucosa model

The assembly of the three‐dimensional peri‐implant mucosa models had duration of 25 days. Briefly, a titanium disk (implant material) was integrated into collagen‐embedded human gingival fibroblasts (HGFs). On the top of the fibroblast‐collagen gel, oral keratinocytes (OKF6/TERT‐2) were added around the titanium and differentiated. The morphology of the peri‐implant mucosa model was evaluated by van Gieson staining and immunohistochemistry in order to confirm that the mucosal structure reflected the previously published engineered human oral mucosa (Dongari‐Bagtzoglou & Kashleva, 2006). The organotypic oral mucosa consisted of a differentiated stratified epithelium and the underlying connective tissue, including the HGF. Four different layers of the epithelium, the stratum basale, stratum spinosum, stratum granulosum, and the superficial keratinised layer were seen to be similar to native human gingival tissue (Figure S1A). The suprabasal epithelial layer was stained by cytokeratin 13, the basement membrane by collagen IV, and the keratinized superficial cells by cytokeratin 10 (Figure S1B–D). Sporadic proliferating cells were also detected—mainly at the basal layer—through Ki67 staining (Figure S1E). E‐cadherin and claudin staining confirmed the tight epithelial barrier (Figure S1F–G).

**Figure 2:**
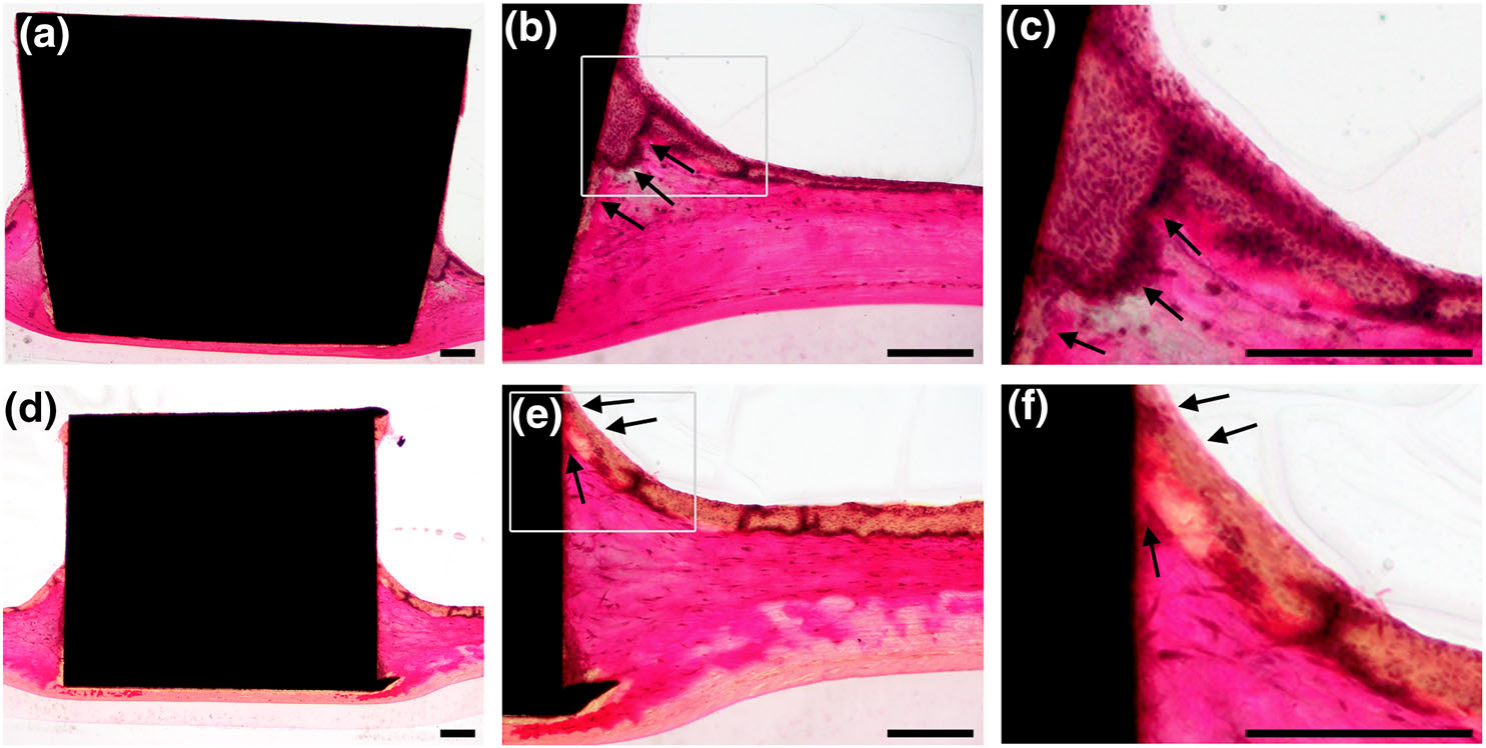
Histological sections of the peri‐implant mucosa model. The inserted implant was integrated into the organotypic mucosa and was non‐colonized or colonized with fibroblasts. Non‐colonized integrated implant: (a) overview of the mucosa‐implant interface, (b), (c) right site at higher magnifications. Fibroblast‐colonized integrated implant: (d) overview of the mucosa‐implant interface, (e), (f) right site at higher magnifications. Arrows indicate epithelial layer growth at the implant‐mucosa interface. The ground sections were stained according to van Gieson. Representative pictures of three independent experiments. Scale bars: 200 μm

The organotypic oral mucosa was attached to the implant and created an intact implant‐mucosa interface (Figure 2). If titanium disks free of fibroblasts were inserted, the epithelial cells grew apically along the titanium disk, deep into the collagen. This apical epithelial migration created an elongated junctional epithelium covering a considerable area of the implant surface (Figure 2a–c). In contrast, the use of fibroblast‐colonized titanium disks hindered such deep epithelial cell migration into the collagen (Figure 2d–f). The staining at the upper part of the implant is related to the fibroblasts, which grew around it prior to insertion into the tissue. The peri‐implant mucosa model with a fibroblast‐colonized titanium disk built an intact mucosa‐implant interface, with only minimal apical epithelial migration along the titanium disk and was used for the following co‐cultures.

### 2.2 S. oralis and A. actinomycetemcomitans biofilms formation

**Figure 3:**
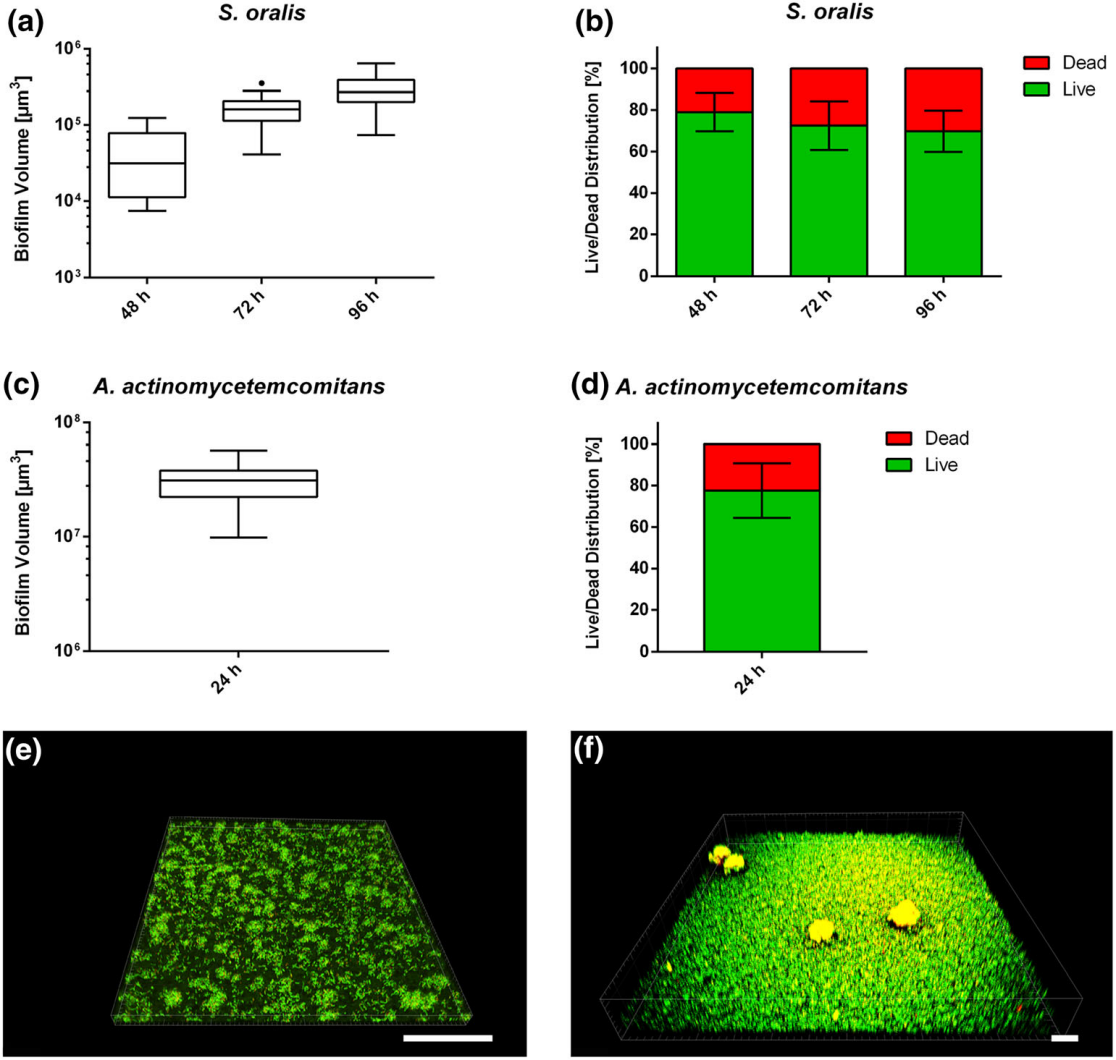
Biofilm formation on supporting material. (a) The Box and Whiskers graphs with Tukey error bars show the total biofilm volumes for Streptococcus oralis after 48, 72, or 96 hr of culture. (b) The bar graphs show the percentages of live and dead bacteria within the S. oralis biofilm. Data of three independent experiments were used for the S. oralis biofilm volume and live/dead distribution. (c) The Box and Whiskers graph with Tukey error bars shows the total biofilm volume for Aggregatibacter actinomycetemcomitans. (d) The bar graph shows the percentages of live and dead bacteria within the A. actinomycetemcomitans biofilm. Data of two independent experiments were used for the A. actinomycetemcomitans biofilm volume and live/dead distribution. (e) Representative 3D image of three independent experiments demonstrating the S. oralis biofilm after 72 hr culture. (f) Representative 3D image of two independent experiments demonstrating the A. actinomycetemcomitans biofilm after 24 hr culture. Live bacteria are depicted in green and dead in red. Scale bars: 100 μm

The developed peri‐implant mucosa should be challenged with either *S. oralis* or *A. actinomycetemcomitans* cells grown as biofilms. Therefore, reproducible and viable sessile communities of these two species were required. Three incubation times were tested for *S. oralis* biofilms: 48, 72, and 96 hr. The biofilm volume, determined by live/dead staining and confocal scanning laser microscopy (CLSM), increased over time, with the maximum detected after 96 hr (Figure 3a). However, at this time point, the proportion of dead bacteria was the highest (Figure 3b). To balance the biofilm volume and viability, we chose the 72‐hr *S. oralis* biofilm (Figure 3e) for the following co‐culture experiments. A viable and thick biofilm of *A. actinomycetemcomitans* was formed on the supporting material after 24 hr of culture (Figure 3c–d). Both cell morphotypes, fimbriated, and non‐fimbriated (corresponding to the rough and smooth colony morphotypes, respectively) were visible in the *A. actinomycetemcomitans* biofilm (Figure 3f).

### 2.3 Histology of the peri‐implant mucosa after biofilm challenge

**Figure 4:**
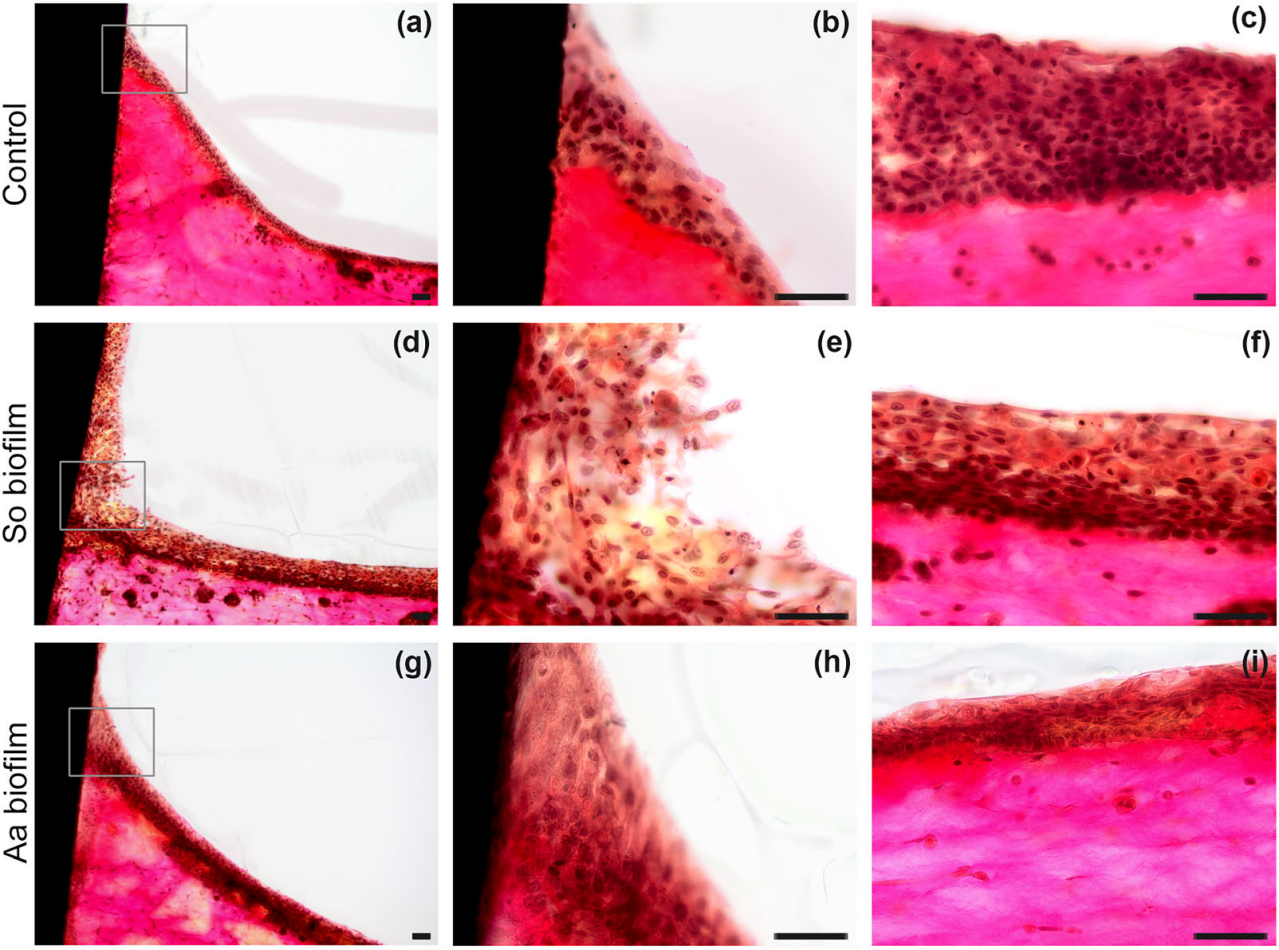
Histological sections of the peri‐implant mucosa‐biofilm model after 24 hr. An overview of the implant‐mucosa interface is shown for the control (a), Streptococcus oralis (d) and Aggregatibacter actinomycetemcomitans (g) biofilm challenged groups. An intact implant‐mucosa interface was observed in the control (b) at higher magnification. The epithelium at the implant site was slightly loosed, after S. oralis biofilm challenge (e), whereas an intact implant‐mucosa interface was observed after co‐culture with the A. actinomycetemcomitans biofilm (h). The adjacent tissues of the control (c), S. oralis (f) and A. actinomycetemcomitans (i) biofilm challenged group were intact. The ground sections were stained according to van Gieson. Representative pictures of three independent experiments. So = S. oralis and Aa = A. actinomycetemcomitans. Scale bars: 50 μm

Peri‐implant mucosa was exposed to either *S. oralis* or *A. actinomycetemcomitans* biofilm for 24 hr, and the effect was evaluated with histological analysis. Co‐cultures with the biofilms resulted in an intact implant‐mucosa interface (Figure 4d,g). The epithelium located directly at the implant was slightly loosened after challenge with the *S. oralis* biofilm (Figure 4d,e). In contrast, the epithelium at a distance from the implant was histologically similar (Figure 4f) to the control tissue (Figure 4a–c). Thus, the effect of the *S. oralis* biofilm was restricted to the implant‐mucosa interface. Exposure to the *A. actinomycetemcomitans* biofilm (Figure 4g–i) had no visible histological effect on the mucosa. Immunohistological staining for adherent junctions (E‐cadherin) and proinflammatory factors (IL‐6, CXCL8, and TNF‐α) was similar for the control and for a tissue exposed to the *S. oralis* biofilm. However, the intensity of claudin staining for tight junctions appeared slightly diminished after co‐culture with the *S. oralis* biofilm (Figure S2).

### 2.4 Transcriptional response of the peri‐implant mucosa to biofilms

**Figure 5:**
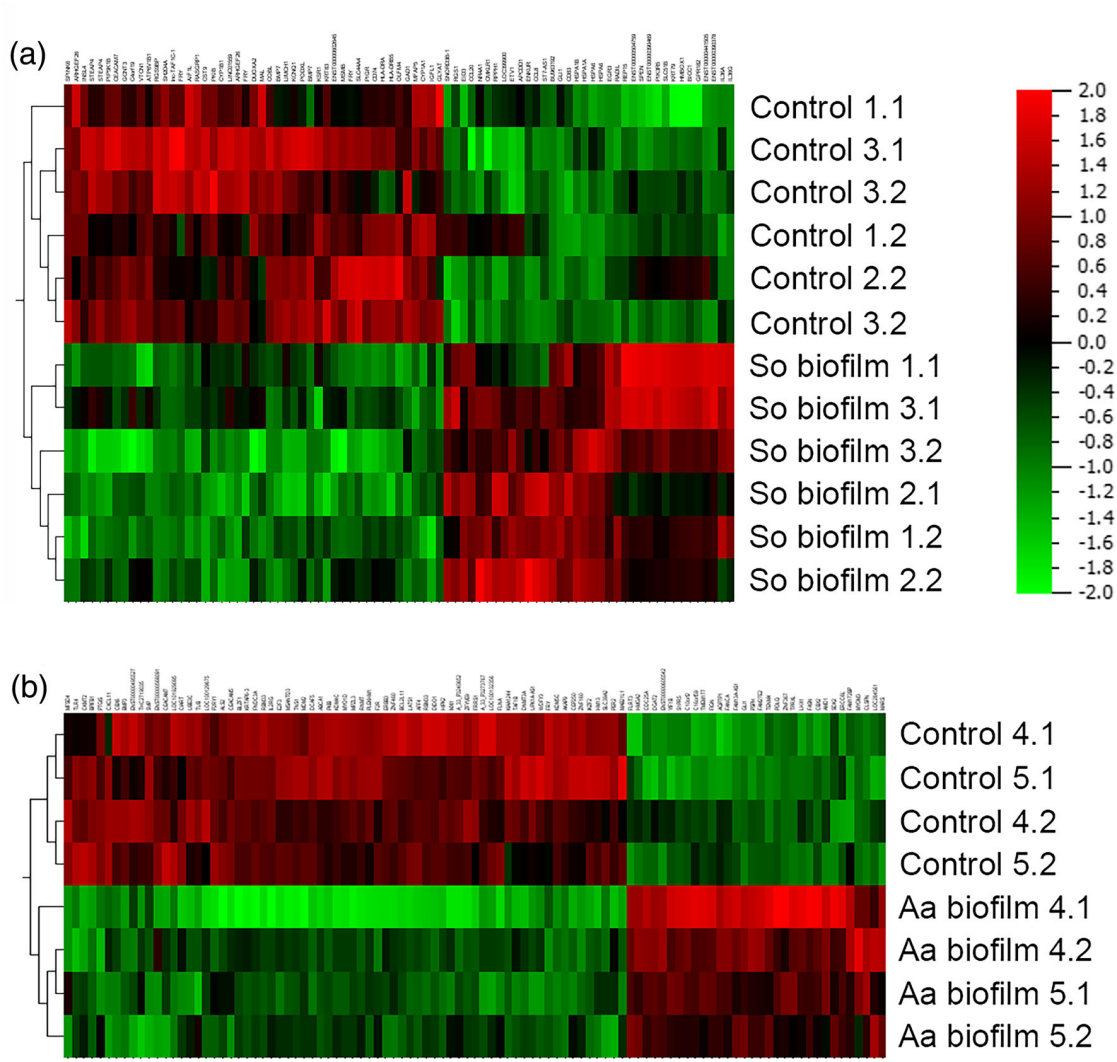
Heat maps of the global gene expression profiles from the peri‐implant mucosa comparing the control and biofilm challenged groups after 24 hr. Results for the Streptococcus oralis (a) and the Aggregatibacter actinomycetemcomitans (b) biofilm are shown in separate heat maps. The heat maps show the hierarchical clustering of the experimental groups and the differentially regulated genes. Data from two‐three independent experiments and duplicates were used. Red indicates upregulation and green downregulation after biofilm co‐culture. So = S. oralis and Aa = A. actinomycetemcomitans

Transcriptional activity of the mucosa was measured by microarrays after 24 hr exposure to biofilms. After co‐culture with the *S. oralis* biofilm, 83 genes were differentially expressed in the peri‐implant mucosa compared with the unexposed tissue. Thirty six genes were upregulated whereas 47 genes were downregulated (Figure 5a). Most of the upregulated genes belonged to the heat‐shock proteins 70 (HSP70). These genes are involved in mitogen‐activated protein kinase signalling and antigen processing and presentation pathways (Table 1). In addition, some genes from the chemokine signalling pathway (i.e., *CCL20*, *CCL8*, and *PIK3R5*) were upregulated. Genes coding for the invariant alpha chain HLA‐DRA of the major histocompatibility complex Class II were downregulated (Table 2). Major histocompatibility complex Class II can be induced by IFN‐γ and is involved in antigen processing and presentation. Challenge of the peri‐implant mucosa with the *A. actinomycetemcomitans* biofilm led to regulation of 101 genes: 32 upregulated and 69 downregulated (Figure 5b). Upregulated genes were related either to cell division (*FIGN, HMGA2, CDC25A*, and *ERCC6L*) or to DNA repair/damage (*CLSPN, POLQ,* and *FANCA*; Table S3). No particular pathway was upregulated. The pathway analysis of the downregulated genes revealed the PI3K‐Akt signalling pathway (Table 3), including genes related to this signal transduction (*MDM2, IL2RG, TLR4,* and *F2R)*.

**Table 1:** Enriched and relevant pathways of upregulated genes in the peri‐implant mucosa‐biofilm model after 24‐hr Streptococcus oralis biofilm challenge

Pathway	%	P	Genes
Antigen processing and presentation	10	.006212	HSPA6, HSPA1A, HSPA1B
MAPK signalling pathway	13.33333	.006514	HSPA6, NR4A1, HSPA1A, HSPA1B
Spliceosome	10	.018203	HSPA6, HSPA1A, HSPA1B
Protein processing in endoplasmic reticulum	10	.028529	HSPA6, HSPA1A, HSPA1B
Chemokine signalling pathway	10	.034067	CCL20, CCL8, PIK3R5

**Table 2:** Enriched and relevant pathways of downregulated genes in the peri‐implant mucosa‐biofilm model after 24‐hr Streptococcus oralis biofilm challenge

Pathway	%	P	Genes
Intestinal immune network for IgA production	7.5	.006521	HLA‐DRB5, PIGR, HLA‐DRA
Antigen processing and presentation	7.5	.016438	HLA‐DRB5, CD74, HLA‐DRA
Cell adhesion molecules (CAMs)	7.5	.052171	VTCN1, HLA‐DRB5, HLA‐DRA
Phagosome	7.5	.057529	HLA‐DRB5, ATP6V1B1, HLA‐DRA
Tryptophan metabolism	5	.099767	CYP1B1, CYP1A1

**Table 3:** Enrichment and relevant pathways of downregulated genes in the peri‐implant mucosa‐biofilm model after 24‐hr Aggregatibacter actinomycetemcomitans biofilm challenge

Pathway	%	P	Genes
PI3K‐Akt signalling pathway	5.434783	.060915	MDM2, IL2RG, TLR4, BCL2L11, F2R

### 2.5 Cytokine secretion

**Figure 6:**
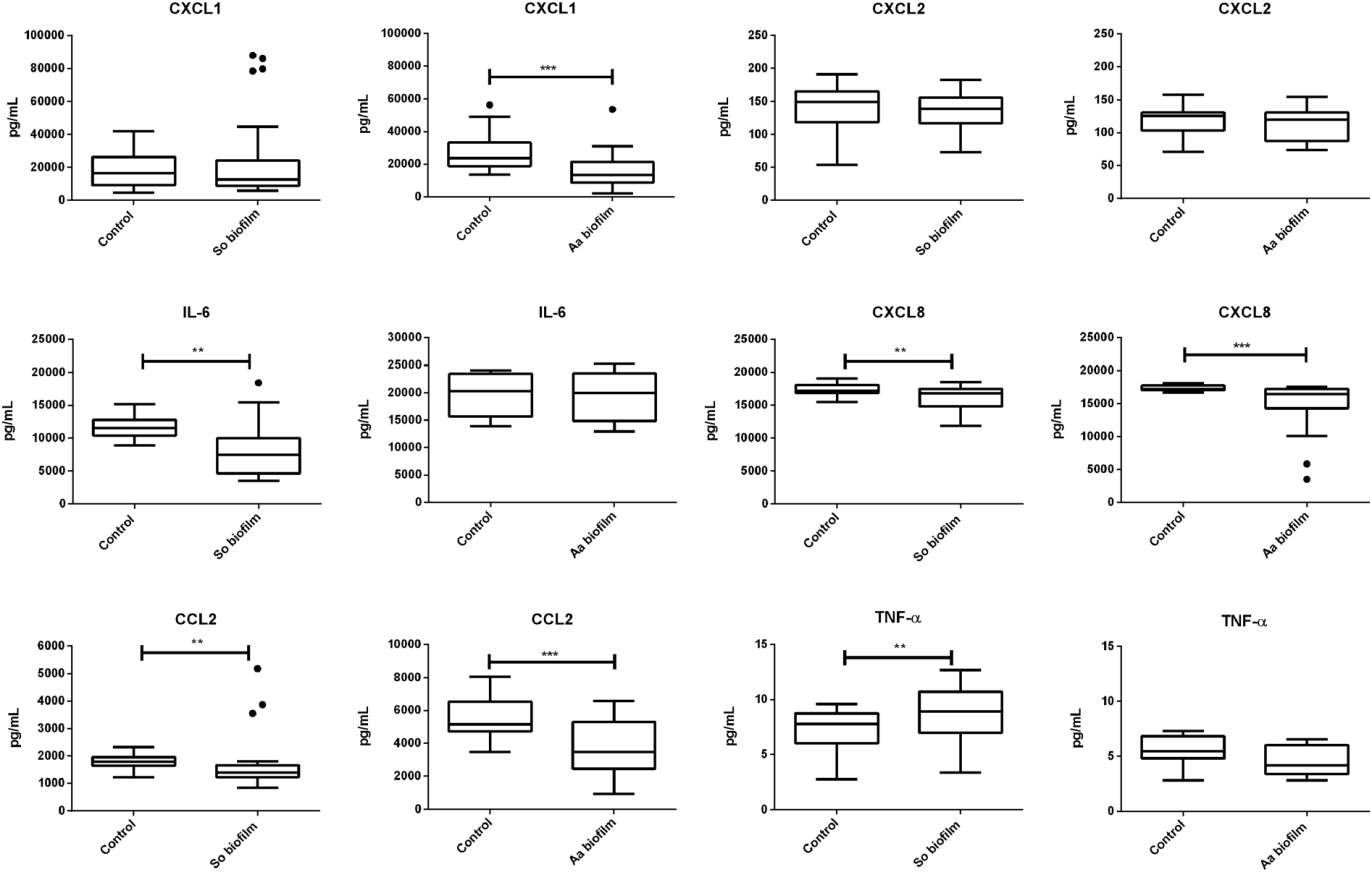
Cytokine levels in the co‐culture supernatants of the peri‐implant mucosa‐biofilm model after 24 hr. The groups challenged with the Streptococcus oralis or Aggregatibacter actinomycetemcomitans biofilm were compared with the control groups for their CXCL1, CXCL2, IL‐6, CXCL8, CCL2, and TNF‐α levels. The cytokines were measured using the luminex technology and a Bio‐Plex Kit. The Box and Whiskers graphs with Tukey error bars represent the measured data points. The S. oralis biofilm group includes 42 measured data points from 14 samples and four independent experiments. The A. actinomycetemcomitans biofilm group includes 29 measured data points from seven samples and two independent experiments. So = S. oralis and Aa = A. actinomycetemcomitans. The statistical significance was determined using the Mann–Whitney method, with P = .05. Single asterisk indicates P < .05 and double asterisk P ≤ .01

The cytokine levels in the collected supernatants were measured by using a Luminex‐based multiplex assay. The results showed that *S. oralis* biofilm challenge caused a significant increase in TNF‐α level in the peri‐implant mucosa compared with the unchallenged tissue (Figure 6). In contrast, the levels of IL‐6, CXCL8, and CCL2 were significantly reduced after stimulation with the *S. oralis* biofilm. Challenge of the peri‐implant mucosa with the *A. actinomycetemcomitans* biofilm led to significant lower levels of CXCL1, CXCL8, and CCL2 (Figure 6). The secretion level of CXCL2 was not affected by any of the studied biofilms.

## 3 DISCUSSION

Peri‐implantitis is a disease with high prevalence (Dreyer et al., 2018). Development of a successful prevention or therapy strategy requires comprehensive understanding of the host–microbe interactions at the peri‐implant site. Here, by applying an organotypic model, we investigated the impact of either commensal *S. oralis* or the pathogenic *A. actinomycetemcomitans* biofilms on the human mucosa. An *in* *vitro* model, which reflects well the clinical situation including the three main compartments, human soft‐tissue, biofilm, and implant material, has been missing until now, and we addressed this problem with our peri‐implant mucosa‐biofilm model (Figure 1). The colonization of the implants with HGF hindered the intensive apical epithelial migration along the implant and thereby the elongated junctional epithelium covering most of the implant surface (Figure 2a–c). As a result, we obtained an organotypic oral mucosa attached to the implant with minimal apical epithelial growth (Figure 2d–f). Intensive apical epithelial migration is a characteristic during severe oral inflammation (Pollanen, Laine, Ihalin, & Uitto, 2012). The resulting implant‐mucosa interface reflected the *in* *vivo* histology and physiology of the healthy mucosa attached to the implant (Atsuta et al., 2005; Schupbach & Glauser, 2007).

The *S. oralis* biofilm led to a slight tissue loosening, which was restricted to the mucosa‐implant interface (Figure 4d,e). Claudin expression seemed to be less after co‐culture with *S. oralis* biofilm (Figure S2). Bacterial stimulation modified tight junctions in lungs, without major disruption of the epithelial barrier, and this correlated with transmigration of polymorphonuclear neutrophils (PMNs; Chun & Prince, 2009). Tissue loosing might facilitate a fast transmigration of PMNs to tissue‐biofilm interface and build a barrier against microbial invasion controlling the bacterial load (Darveau, 2009; G. Hajishengallis & Lamont, 2016). Our model could benefit from future implementation of PMNs to study their transmigration and bacterial clearance. In contrast to *S. oralis*, the exposure to *A. actinomycetemcomitans* biofilm did not disturb the mucosa (Figure 4g–i), which was also observed by others (T. Ahlstrand et al., 2017; A. Paino et al., 2012). Probably the *S. oralis* biofilm supports the immune cell migration through tissue loosening, in contrast to the *A. actinomycetemcomitans* biofilm that has no impact on the tissue structure.

Transcriptional analysis revealed a broader gene response to the *A. actinomycetemcomitans* biofilm compared with the *S. oralis* biofilm (Figure 5). Hasegawa *et* *al*. compared the transcriptional response of keratinocytes with commensals or opportunistic pathogens and detected similar differences as reported here (Handfield et al., 2005; Hasegawa et al., 2007). The weak transcriptional response to commensal bacteria supports the adaptive coevolution theory of commensal bacteria with the oral mucosa (Handfield, Baker, & Lamont, 2008; Hooper & Gordon, 2001). The overall response to *S. oralis* at the transcriptional level was related to protective response. Pathways related to tissue protection were upregulated (Table 1) including *CCL20* and genes grouped in HSP70*,* with functions in mucosal homeostasis (Comerford et al., 2010; Pleguezuelos, Dainty, Kapas, & Taylor, 2005; Schutyser, Struyf, & Van Damme, 2003). In addition, the adaptive immune response was suppressed as indicated by the downregulation of antigen presentation and processing (Table 2). These might lead to a state of unresponsiveness—with decreased both humoral and cell‐mediated immune response (Han et al., 2003; Hasegawa et al., 2007). Hyporesponsiveness induced by commensals probably plays a role in protection from tissue destruction induced by inflammatory response (Pollanen et al., 2012). Compared with the *S*. *oralis* biofilm, transcriptional response to *A*. *actinomycetemcomitans* was broader without targeting pathways. Upregulated genes were related to DNA damage, DNA repair, and cell division suggesting general stress response. Analysis of the downregulated genes revealed a single enriched pathway: the PI3K‐Akt signalling (Table 3). Attenuation of this pathway by *P*. *gingivalis* can promote its invasion and colonisation of the mucosal tissue (Nakayama, Inoue, Naito, Nakayama, & Ohara, 2015). Similarly, our observed changes may promote colonization and survival of *A*. *actinomycetemcomitans*. In summary, the transcriptional profiles of the peri‐implant mucosa revealed a tissue protective response to the *S*. *oralis* biofilm and a stress response to the *A*. *actinomycetemcomitans* biofilm.

The classical proinflammatory cytokine IL‐6 and the neutrophil recruiting chemokines CXCL8 and CCL2 were found at lower levels in the supernatants after challenge with the *S. oralis* biofilm (Figure 6). Corresponding to our results, different studies could show that commensal bacteria reduce the proinflammatory cytokines, IL‐6 and CXCL8 (Cosseau et al., 2008; Hasegawa et al., 2007; Twetman et al., 2009; Zhang, Chen, & Rudney, 2008). Therefore, *S. oralis* biofilm might attenuate the proinflammatory response, which is consistent with our observations on gene expression. TNF‐α was increased in response to the *S. oralis* biofilm (Figure 6). It is one of the main inflammation mediators (Groeger & Meyle, 2015) and is present at low levels in the gingival crevicular fluid in healthy patients (Darveau, 2010; Petkovic‐Curcin, Matic, Vojvodic, Stamatovic, & Todorovic, 2011). Probably, cytokines controlled by commensal bacteria are involved in limiting biofilm development and consequently in maintaining gingival health (Darveau, 2010; Dickinson et al., 2011; Rouabhia, 2002). After challenge with the *A. actinomycetemcomitans* biofilm, the levels of CXCL1, CXCL8, and CCL2 were lower than in the control (Figure 6). Previously, it was found that *A. actinomycetemcomitans* can sense and bind cytokines; among them was CXCL8 (T. Ahlstrand et al., 2017; T. Ahlstrand et al., 2018; T. Ahlstrand, Kovesjoki, Maula, Oscarsson, & Ihalin, 2019). Lower metabolic activity of biofilms induced by cytokine binding could lead to higher resistance (A. Paino et al., 2011) and could reduce the production of virulence factors providing an explanation why the peri‐implant mucosa was not impaired after challenge with the *A. actinomycetemcomitans* biofilm. The lower levels of chemotactic cytokines may reduce immune cell recruitment leading to a colonization advantage by *A. actinomycetemcomitans*. Interleukin depletion may act in concert with virulence factors, like adhesion and toxins, and compromise tissue integrity at later infection (Henderson, Ward, & Ready, 2010; Szafranski et al., 2017). In summary, the *S. oralis* biofilm attenuated the proinflammatory response of the peri‐implant mucosa; nevertheless, basic awareness was maintained through increased TNF‐α level. On the contrary, *A. actinomycetemcomitans* diminished proinflammatory response creating a colonization advantage and potentially facilitates biofilm expansion.

The *in* *vitro* peri‐implant mucosa‐biofilm model reflected the local response of the host to the biofilms. The *in* *vivo* host–microbe interactions include also immune cells (Pollanen et al., 2012), which were not present in our model. However, our results are in line with *in* *vivo* studies, which showed that commensals and pathogens alone do not induce inflammation, in contrast to their co‐infections (Diaz et al., 2012; Ramsey & Whiteley, 2009; Whitmore & Lamont, 2011; Xu et al., 2014) suggesting that microbial synergy plays an important role in the pathogenesis. Within the limitations of our study, responses on transcription and cytokine levels were uncovered that may explain why monospecies commensal and pathogenic biofilms do not cause inflammatory response. Noteworthy, the reactions from the peri‐implant mucosa to these monospecies biofilms were in accordance with previous *in* *vivo* observations.

In conclusion, our novel peri‐implant mucosa‐biofilm model promises enormous experimental potential to investigate the interaction of three key components: mucosa, biofilm, and implant. Our 3D model reflected that commensal streptococci induce a balanced immune response of the soft tissue including specific transcriptional response and attenuated pro‐inflammatory cytokines. This subtle effect could preserve the oral health. Furthermore, the colonization advantage of opportunistic pathogens by suppression of inflammatory reaction could favour dysbiosis. We showed that species‐specific molecular reactions of the peri‐implant mucosa to biofilm can be successfully studied in our peri‐implant mucosa‐biofilm model.

Our model permits future investigations of health‐related or dysbiotic multispecies biofilms as well as phages, bacterial viruses (Preus, Olsen, & Namork, 1987; Szafranski, Winkel, & Stiesch, 2017). The influence of various implant materials and surface functionalisation on biofilm formation and tissue reaction are additional factors that will be analyzed in the future. Accordingly, the findings will provide new opportunities for future strategies of disease prevention and treatment as well as for implant improvement.

## 4 EXPERIMENTAL PROCEDURES

### 4.1 Cell culture

HGFs (121 0412, Provitro GmbH) were cultured in Dulbecco's modified Eagle's medium (DMEM, FG0435, Biochrom AG), supplemented with 10% fetal bovine serum (FBS, P30‐3309, PAN‐Biotech GmbH), 100‐U/ml penicillin, and 100‐μg/ml streptomycin (A2212; Biochrom AG). The immortalized human oral keratinocyte cell line (OKF6/TERT‐2; Dickson et al., 2000) was cultured in KerSFM medium (10725‐018, Gibco Lifetechnologies), supplemented with 0.3‐mM CaCl_2_, 0.2‐mg/ml EGF, 25‐μg/ml BPE, 100‐U/ml penicillin, and 100‐μg/ml streptomycin. The cells were incubated at 37°C in a 5% CO_2_ humidified atmosphere.

### 4.2 Peri‐implant mucosa model

The peri‐implant oral mucosa model assembly was based on the protocol of Dongari‐Bagtzoglou and Kashleva (Dongari‐Bagtzoglou & Kashleva, 2006) and reproduced with slight modifications. Briefly, bovine type I collagen (2‐mg/ml PureCol®, 5005‐100ML, Advance Biomatrix) was mixed with FBS, L‐glutamine (G7513, Sigma‐Aldrich), 10 x DMEM (P03–01510, Pan‐Biotech), and a DMEM reconstitution buffer (2.2‐mg/ml sodium bicarbonate, 2‐mM HEPES, and 0.0062 N NaOH in DMEM P03‐01510). HGFs (passage 9 or 10) were then added to the collagen mixture and poured into culture inserts (PIHA 03050, Merck Millipore or 3414, Corning B.V. Life Sciences). Each model contained 4 × 10^5^ HGFs in the collagen. Titanium disks (3 mm diameter, 2.3 mm height, Grade 4, machined surface) were colonized with HGF (1 × 10^6^ cells/ml). Both were cultivated and submerged in fully supplemented DMEM at 37°C in a humidified atmosphere with 5% CO_2_. At day 5, a titanium disk with or without HGF colonization was integrated. For this purpose, the models were punched with a 2.5 mm diameter biopsy punch. The titanium disks were placed in the resulting holes. After 3 days, 1.2 × 10^6^ oral keratinocytes (OKF6/TERT‐2, Passages 19–26) were seeded on the top of each fibroblast‐collagen gel. At day 12, the models were raised to an air‐liquid interface and cultivated for 13 days with a specific Airlift (AL) medium (3:1 DMEM [P04‐03591, Pan‐Biotech] and Ham's F‐12 [P04‐14559, Pan‐Biotech], 5‐μg/ml insulin, 0.4‐μg/ml hydrocortisone, 2 × 10^−11^ M 5‐triiodo‐L‐thyronine, 1.8 × 10^−5^ M adenine, 5‐μg/ml transferrin, 10^−10^ M cholera toxin, 2 mM L‐glutamine, 10% v/v FBS, 1% v/v penicillin/streptomycin).

### 4.3 Biofilm formation and quantification

*S. oralis* (DSM 20627, German collection of microorganisms and tissue culture cells) was cultivated in tryptone soya broth supplemented with 10% yeast extract (TSBY) for 18 hr under stirring at 37°C in an anaerobic atmosphere. For biofilm formation, *S. oralis* was diluted in Brain Heart Infusion (BHI) supplemented with 5% w/v sucrose to an optical density (600 nm) of 0.06, corresponding to 8.7 × 10^7^ CFU/ml. The biofilm was cultured on a hydrophilic polyethersulfone membrane (GPWP04700, Merck Millipore) for 48, 72, or 96 hr at 37°C in a humidified atmosphere with 5% CO_2_. The medium was replaced each day with fully supplemented BHI. *A*. *actinomycetemcomitans* JP2 strain (HK1651, CCUG 56173, Culture Collection, University of Göteborg) isolated from aggressive juvenile periodontitis was cultivated in Todd‐Hewitt broth supplemented with 10% yeast extract (THBY) for 72 hr at 37°C in a humidified atmosphere with 5% CO_2_. Smooth and rough colony morphotypes were isolated and cultured separately. For biofilm formation, the *A. actinomycetemcomitans* strain was diluted in THBY to an optical density (600 nm) of 0.6 for smooth and 0.05 for rough type, corresponding to 3.25 × 10^7^ CFU/ml and 2.3 × 10^6^ CFU/ml. The two *A. actinomycetemcomitans* morphotypes were mixed at equal volumes and cultured on glass coverslips (18 mm diameter, CBAD00180RA120MNZ#0, Thermo Scientific Menzel) for 24 hr at 37°C in a humidified atmosphere with 5% CO_2_, to build a biofilm.

The *S. oralis* or *A. actinomycetemcomitans* biofilms, which were formed on the supporting material, were stained with the LIVE/DEAD®BacLight™ Bacterial Viability Kit (L7012, Life Technologies). After phosphate buffered saline (PBS) wash, the biofilms were fluorescently stained with SYTO9 and propidium iodide as a 1:1,000 dilution in PBS for 30 min. The biofilms were washed with PBS and subsequently fixed in 2.5% glutardialdehyde in PBS. The stained *S. oralis* biofilms were imaged at 40‐fold magnification through CLSM (Leica TCS SP2). Three random positions were scanned by creating z‐plane images. The stained *A. actinomycetemcomitans* biofilms were imaged at 10‐fold magnification through CLSM (Leica TCS SP8). Five random positions were scanned by creating z‐plane images. For both biofilms, 3D images were reconstructed by the Imaris® × 64 6.2.1 software package (Bitplane) and used to calculate the volume in the surpass mode. Finally, the percentages of live and dead cells were calculated.

### 4.4 Co‐culture of the peri‐implant mucosa with the biofilms

The co‐cultures were conducted in AL‐medium without any antibiotics. The peri‐implant oral mucosa model was used and washed with PBS prior to co‐culture. Either the 72‐hr‐old *S. oralis* or *A. actinomycetemcomitans* biofilm was washed five times with PBS and was placed on spacers with the biofilm side facing the peri‐implant oral mucosa model with direct contact to the integrated titanium disk (Figure 1). The co‐cultures were performed for 24 hr at 37°C in a 5% CO_2_ humidified atmosphere.

### 4.5 RNA extraction and microarray data analysis

The supernatants were collected after co‐culture for subsequent analysis of the secreted cytokines. The tissues were stored in RNAlater™ Solution (AM7020, Invitrogen) overnight at 4°C. Tissue RNA was isolated according to the manufacturer's protocol using the RNeasy® Mini Kit (74104, Qiagen). Briefly, the tissues were lysed with a microcentrifuge pestle in RLT buffer with 1% v/v β‐mercaptoethanol and vortexed. The lysates were homogenized using the QIAshredder (79654, Qiagen), and the RNA was isolated by using the RNeasy® Mini Kit (74104, Qiagen). The RNA was stored at −80°C for later analysis.

For the gene expression analysis, total RNA was reverse‐transcribed into double strand cDNA, and the Cy3‐labelled cRNA was synthesized by using the Quick Amp Labeling Kit, One Colour (Agilent) according to the manufacturer's instructions. The cRNA was purified with the RNeasy® Mini Kit (Qiagen). For cRNA fragmentation, hybridization, and washing, the One‐Colour Microarray‐Based Gene Expression Analysis Protocol V5.7 (Agilent) was used. The Cy3‐labelled cRNA (2,500 ng) was hybridized on the refined 026652QM_RCUG_HomoSapiens microarray (34,127 genes), which was developed at the Research Core Unit Genomics of the Hannover Medical School, for 17 hr at 65°C. The Agilent Micro Array Scanner G2565CA was used for scanning the slides. The raw data were extracted with the Feature Extraction Software V10.7.3.1 (pixel resolution 3 μm, bit depth 20) and imported into Qlucore Omics Explorer software under default import settings for Agilent One‐Colour Microarray for further transcriptomic analysis. Biofilm‐challenged and control tissues were compared with Student's *t* test under the conditions of log2 ratio > 2 and *P* < .01. The false discovery rate for tissues challenged with *S. oralis* and *A. actinomycetemcomitans* biofilm were *q* = 0.009321 and *q* = 0.408989, respectively.

Pathway analysis was performed by DAVID (Database for Annotation, Visualisation and Integrated Discovery; Huang da, Huang, Sherman, & Lempicki, 2009a; Huang da, Huang, Sherman, & Lempicki, 2009b) using default settings for the upregulated and downregulated gene lists (Table S1 and S2) after biofilm challenge, which were analyzed separately.

### 4.6 Detection of cytokines in the supernatant

Cytokine (CXCL1, CXCL2, IL‐6, CXCL8, CCL2, and TNF‐α) quantification was performed using a customized all in one Bio‐Plex Pro Human Chemokine 6plx EXP kit (17002259, Bio‐Rad). The cytokines in the collected supernatants were measured by the Luminex‐based multiplex technique according to the manufacturer's instructions (Bio‐Rad). The concentrations were calculated with Bio‐Plex Manager 6.0 by comparison with the standard curves. The detection sensitivity ranged between 1 pg and 40 μg of protein per 1 ml.

### 4.7 Histological analysis

The peri‐implant oral mucosa models were fixed in a 4% buffered formalin solution for 24 hr. The samples were watered, dehydrated by using an ethanol gradient, and embedded in Technovit 9100. The embedded samples were either cut into 5‐μm slides for implant‐free sections or were grinded to 22–36‐μm slides for the peri‐implant ground sections according to the cutting‐grinding technique by Donath K. Prior staining, the Technovit 9100 was removed by rinsing the slides in acetone. Afterwards, the slides were rehydrated by using an ethanol gradient. Finally, the slides were stained according to van Gieson or specific antibodies. For van Gieson staining, the slides were rinsed for 10 min in ferric haematoxylin, and then washed once with tap water and twice with hydrochloric acid alcohol. After rinsing in tap water for 10 min, they were added into the van Gieson solution for 3 min, subsequently washed with 96% ethanol, 100% ethanol, xylol, and finally mounted. For immunohistochemical staining, the slides were washed with distilled water, washing buffer, antigen retrieval buffer, and washing buffer. The slides were incubated overnight with the primary antibody. All primary antibodies were against human epitopes. The rabbit polyclonal anti‐claudin 1 (359‐14), mouse monoclonal anti‐collagen Type IV CIV22 (239 M‐15), rabbit monoclonal anti‐cytokeratin 10 EP97 (410R‐14), rabbit monoclonal anti‐cytokeratin 13 EP69 (AC‐0066A), and mouse monoclonal anti‐Ki67 MIB‐1 (ILM 9252 C01) were purchased from medac GmBH. Mouse monoclonal anti‐interleukin 6 (ABIN2469708) and mouse monoclonal anti‐interleukin 8 (ABIN1724413) were purchased from antibodies‐online GmbH. The slides were rinsed in wash buffer, peroxide block, and washing buffer prior to incubation with the corresponding secondary detection antibody. The secondary antibodies, Histofine Simple Stain MAX PO goat anti‐mouse Ig F (ab`)‐fragments (414131F) and Histofine Simple Stain MAX PO goat anti‐rabbit Ig F (ab`)‐fragments (414141F) were purchased from medac GmBH. The slides were then rinsed in washing buffer, incubated for 10 min with DAB (957D‐50) and washed with distilled water. They were counterstained in haematoxylin (Leica), rinsed in distilled water, dehydrated using an ethanol gradient, washed with xylol, and mounted. All slides were examined under the Olympus CX41 microscope.

### 4.8 Statistical analysis

All presented data were derived from two to three independent experiments.

Statistical evaluation of the cytokine levels was performed using GraphPad Prism 7. A Mann–Whitney test was used to analyze the statistical differences between the controls and biofilm groups. Differences were considered statistically significant at *P* < .05.

## CONFLICT OF INTEREST

The authors declare no conflict of interest.

## Supporting information

Data S1: Supplementary Information
