# Supplementary material for: Commensal and pathogenic biofilms differently modulate peri‐implant oral mucosa in an organotypic model
Source: Cell Microbiol. 2019 Jul 17;21(10):e13078. doi: 10.1111/cmi.13078 (PMC6771885; doi:10.1111/cmi.13078)
Supplement: Supplementary file 1 — Data S1: Supplementary Information [file CMI-21-na-s001.pdf]

Supplemental Material

**Commensal and pathogenic biofilms differently modulate peri-implant oral mucosa in an organotypic model**

Alexandra Ingendoh-Tsakmakidis^1, 2^, Carina Mikolai^1, 2^, Andreas Winkel^2^, Szymon P. Szafrański^2^, Christine S. Falk^3^, Angela Rossi^4^, Heike Walles^4, 5^, Meike Stiesch^2^

^1^ Equally contributing authors

^2^Department of Prosthetic Dentistry and Biomedical Materials Science, Hannover Medical School, Carl-Neuberg-Str. 1, 30625 Hannover, Germany

^3^Institute of Transplant Immunology, Hannover Medical School, Carl-Neuberg-Str. 1, 30625 Hannover, Germany

^4^Fraunhofer Institute of Silicate Research ISC, Translational Center for Regenerative Therapies, 97070, Würzburg, Germany.

^5^University Hospital of Würzburg, Chair of Tissue Engineering and Regenerative Medicine, 97070, Würzburg, Germany.

Alexandra Ingendoh-Tsakmakidis, Department of Prosthetic Dentistry and Biomedical Materials Science, Stadtfelddamm 34, 30625 Hannover, Germany, Phone: 0049 511 532 1418, [Ingendoh-Tsakmakidis.Alexandra@mh-hannover.de](mailto:Ingendoh-Tsakmakidis.Alexandra@mh-hannover.de)

Carina Mikolai, Department of Prosthetic Dentistry and Biomedical Materials Science, Stadtfelddamm 34, 30625 Hannover, Germany, Phone: 0049 511 532 1417, [Mikolai.Carina@mh-hannover.de](mailto:Mikolai.Carina@mh-hannover.de)

Running title: Host modulation by *S. oralis* and *A. actinomycetemcomitans* in a 3D model


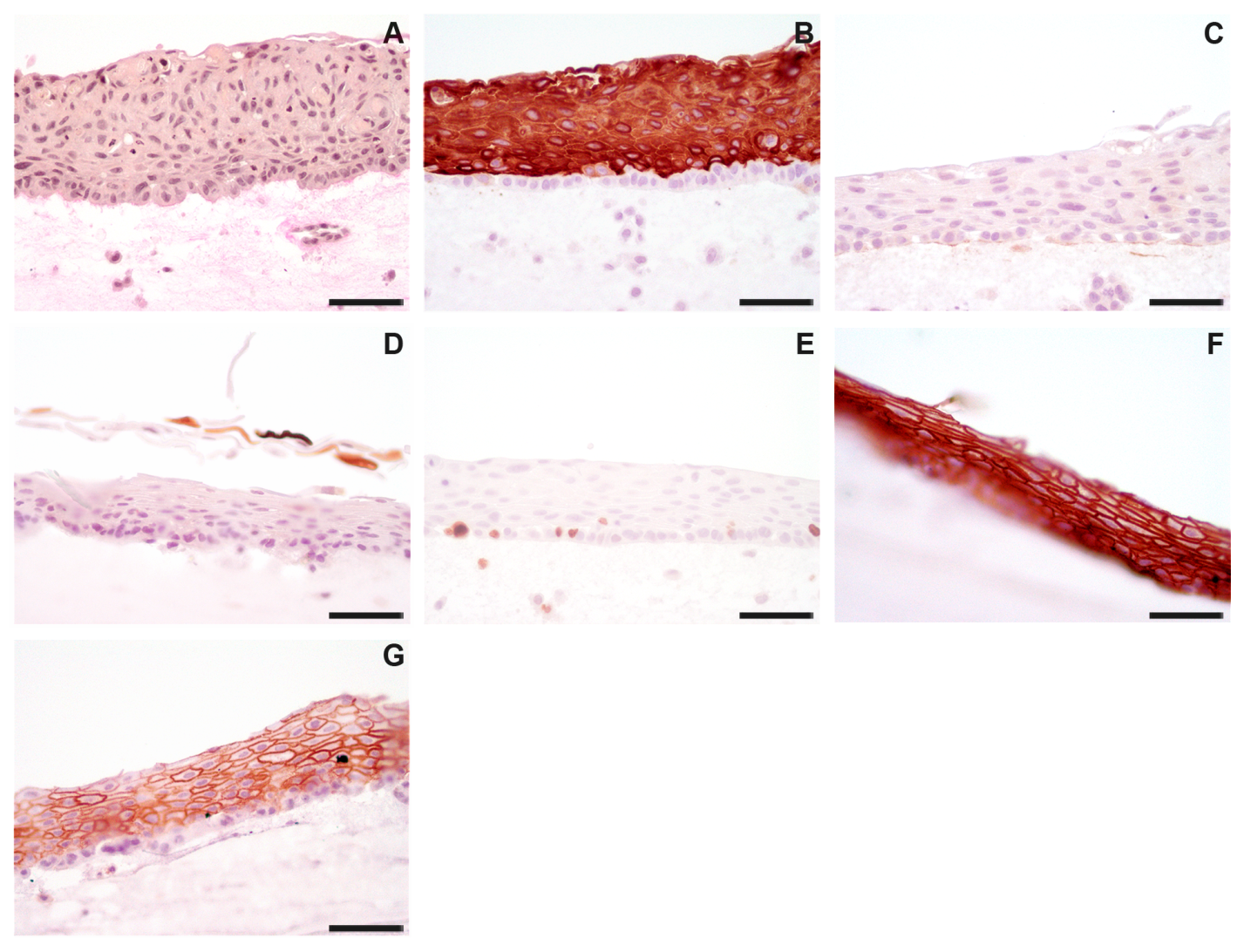


Figure S1. Histological and immunohistological sections of the organotypic oral mucosa. The histological section was stained according to van-Gieson (A). Immunohistochemical staining for cytokeratin 13 (B), collagen IV (C), cytokeratin 10 (D), Ki67 (E), E-cadherin (F), and claudin (G) is depicted in brown. Scale bars: 50 μm.


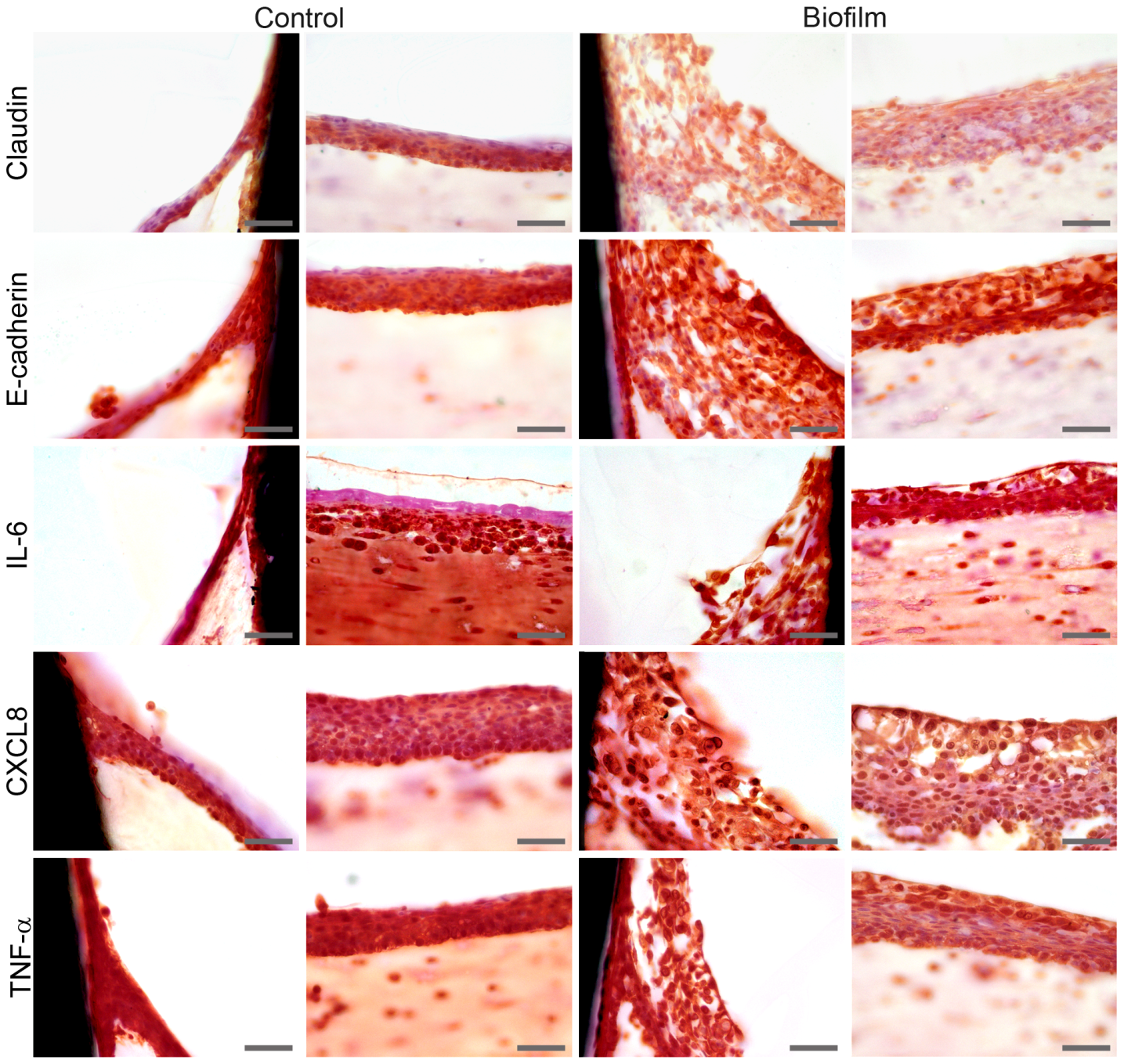


Figure S2. Immunohistochemical staining of the peri-implant mucosa-biofilm model after 24 hours *S. oralis* biofilm challenge. The pictures show the implant-mucosa interface (left) and the adjacent tissue (right). The ground sections were stained for claudin, E-cadherin, IL-6, CXCL8, and TNF-α. Representative pictures of three independent experiments. Scale bars: 50 µm.

Table S1. List of upregulated genes after *S. oralis* biofilm challenge with corresponding fold change and p-value. Biofilm group compared to the control group.

| GeneName | SystematicName | p-value | Fold change |
| --- | --- | --- | --- |
| GLI1 | NM_005269 | 6.70E-06 | 0.409584 |
| HSPA1A | NM_005345 | 1.31E-06 | 0.292796 |
| RADIL | NM_018059 | 6.20E-05 | 0.491592 |
| HSPA6 | NM_002155 | 1.28E-06 | 0.155768 |
| IL36G | NM_019618 | 0.000522 | 0.476491 |
| CCL20 | NM_004591 | 0.000231 | 0.418091 |
| CCL8 | NM_005623 | 0.003166 | 0.1848 |
| EGR3 | NM_004430 | 1.65E-05 | 0.426861 |
| APCDD1 | NM_153000 | 0.009899 | 0.461692 |
| IL36A | NM_014440 | 0.004909 | 0.43665 |
| SLC51B | NM_178859 | 0.00496 | 0.3916 |
| EID3 | NM_001008394 | 0.001171 | 0.465359 |
| PIK3R5 | NM_014308 | 0.004881 | 0.444812 |
| CD83 | NM_004233 | 3.65E-05 | 0.496485 |
| RGS1 | NM_002922 | 0.001105 | 0.485504 |
| BU963192 | BU963192 | 1.60E-08 | 0.119727 |
| CMKLR1 | NM_001142343 | 0.002571 | 0.393855 |
| HMBOX1 | ENST00000397358 | 0.009065 | 0.265799 |
| ST7-AS1 | NR_002330 | 0.005864 | 0.484365 |
| ENST00000390469 | ENST00000390469 | 0.005856 | 0.428607 |
| SNORD3B-1 | NR_003271 | 0.002244 | 0.495459 |
| GPR182 | NM_007264 | 0.009866 | 0.305853 |
| ENST00000504759 | ENST00000504759 | 0.009716 | 0.43867 |
| REP15 | NM_001029874 | 0.001827 | 0.474339 |
| ENST00000390378 | ENST00000390378 | 0.000499 | 0.393867 |
| LOC399900 | XR_247232 | 0.001535 | 0.477012 |
| BICC1 | NM_001080512 | 0.007093 | 0.325945 |
| KRT79 | NM_175834 | 0.006142 | 0.331853 |
| NR4A1 | NM_001202234 | 0.002299 | 0.476577 |
| ENKUR | NM_145010 | 0.007902 | 0.483367 |
| ENST00000441505 | ENST00000441505 | 0.004375 | 0.380711 |
| SPEN | NM_015001 | 0.006174 | 0.378474 |
| ETV1 | NM_004956 | 0.006246 | 0.496457 |
| HSPA6 | NM_002155 | 7.01E-05 | 0.362233 |
| RPPH1 | NR_002312 | 0.002544 | 0.47743 |
| HSPA1B | NM_005346 | 3.06E-05 | 0.430316 |

Table S2. List of downregulated genes after *S. oralis* biofilm challenge with corresponding fold change and p-value. Biofilm group compared to the control group.

| GeneName | SystematicName | p-value | Fold change |
| --- | --- | --- | --- |
|  |  |  |  |
| FRY | NM_023037 | 0.006674 | 2.455937 |
| INSL4 | NM_002195 | 0.000285 | 2.961887 |
| RASGRP1 | NM_005739 | 0.000642 | 2.259549 |
| ATP6V1B1 | NM_001692 | 0.001211 | 2.639741 |
| PKIB | NM_181795 | 0.000661 | 2.023561 |
| CYP1A1 | NM_000499 | 2.00E-05 | 2.586725 |
| SH2D4A | NM_022071 | 1.30E-05 | 2.104773 |
| MAL | NM_002371 | 0.009321 | 3.382023 |
| KSR1 | NM_014238 | 0.000795 | 2.118257 |
| PODXL | NM_001018111 | 0.00257 | 2.227458 |
| C4orf19 | NM_018302 | 0.005837 | 2.046746 |
| GAD1 | NM_000817 | 9.51E-05 | 2.138951 |
| AIF1L | NM_001185095 | 0.000348 | 2.825256 |
| GLYAT | NM_005838 | 0.001932 | 2.356111 |
| GCNT3 | NM_004751 | 0.000129 | 2.15584 |
| KCNQ1 | NM_000218 | 0.000145 | 2.441704 |
| HLA-DRB5 | NM_002125 | 0.004059 | 2.332992 |
| VTCN1 | NM_024626 | 0.00032 | 3.4584 |
| BMP7 | NM_001719 | 8.09E-05 | 2.043681 |
| ARHGEF26 | NM_015595 | 4.86E-05 | 2.064054 |
| CD74 | NM_001025158 | 0.001123 | 3.253644 |
| SPINK6 | NM_205841 | 2.26E-06 | 4.496015 |
| MSMB | NM_002443 | 0.002093 | 2.136284 |
| OLFM4 | NM_006418 | 0.000379 | 4.506128 |
| CEACAM7 | NM_006890 | 3.59E-05 | 2.135743 |
| KRT83 | NM_002282 | 0.000633 | 2.006753 |
| SLC44A4 | NM_025257 | 0.001794 | 2.067554 |
| PIGR | NM_002644 | 0.000281 | 3.266208 |
| BMP7 | NM_001719 | 0.003105 | 2.081317 |
| LIMCH1 | NM_014988 | 0.00275 | 2.066329 |
| RGS9BP | NM_207391 | 0.000101 | 2.124365 |
| PIP5K1B | NM_003558 | 0.000674 | 2.636583 |
| ARHGEF26 | NM_001251962 | 8.33E-05 | 2.261612 |
| LINC01559 | NR_036555 | 0.001481 | 2.281359 |
| CST9 | NM_001008693 | 0.00221 | 2.024063 |
| HLA-DRA | NM_019111 | 0.004796 | 3.167702 |
| ENST00000602645 | ENST00000602645 | 0.000678 | 2.204943 |
| FRY | NM_023037 | 0.000615 | 2.680749 |
| FRY | NM_023037 | 0.003205 | 2.059694 |
| DUOXA2 | NM_207581 | 0.005822 | 2.339642 |
| IGFL1 | NM_198541 | 0.000132 | 2.236622 |
| CYP1B1 | NM_000104 | 0.000274 | 2.021134 |
| STEAP4 | NM_001205315 | 0.000542 | 2.290926 |
| lnc-TAF1C-1 | lnc-TAF1C-1:1 | 0.000375 | 2.081175 |
| STEAP4 | NM_001205315 | 0.00374 | 2.191968 |
| MFAP5 | NM_003480 | 4.97E-05 | 2.4087 |
| COBL | NM_015198 | 1.52E-06 | 3.426142 |

Table S3. List of upregulated genes after *A. actinomycetemcomitans* biofilm challenge with corresponding fold change and p-value. Biofilm group compared to the control group.

| GeneName | SystematicName | p-value | Fold change |
| --- | --- | --- | --- |
| GLI1 | NM_005269 | 0.000258 | 0.4367 |
| S1PR5 | NM_030760 | 0.002052 | 0.423775 |
| CLSPN | NM_022111 | 0.000247 | 0.453492 |
| C10orf2 | NM_021830 | 0.00443 | 0.486977 |
| YIF1B | NM_001145463 | 0.004655 | 0.464984 |
| FANCA | NM_000135 | 0.006542 | 0.497809 |
| POLQ | NM_199420 | 0.008039 | 0.454652 |
| C16orf59 | NM_025108 | 0.009938 | 0.492998 |
| MYOM3 | NM_152372 | 0.008925 | 0.428346 |
| DGAT2 | NM_032564 | 0.00198 | 0.328055 |
| TMEM177 | NM_030577 | 0.00716 | 0.408168 |
| HMGA2 | NM_003483 | 0.001663 | 0.395813 |
| ERCC6L | NM_017669 | 0.004604 | 0.405565 |
| MARS | NM_004990 | 0.003352 | 0.426933 |
| TPRXL | NR_002223 | 0.003155 | 0.463827 |
| TENM4 | NM_001098816 | 0.002543 | 0.458728 |
| AMD1 | NM_001634 | 0.004942 | 0.448182 |
| CDC25A | NM_001789 | 0.001106 | 0.408356 |
| FAM27E2 | NR_103714 | 0.002644 | 0.492152 |
| ZNF367 | NM_153695 | 0.00735 | 0.444674 |
| SDK2 | NM_001144952 | 0.006662 | 0.474166 |
| FIGN | ENST00000333129 | 0.002642 | 0.463369 |
| PSPH | NM_004577 | 0.002056 | 0.4656 |
| ENST00000605542 | ENST00000605542 | 0.000734 | 0.072871 |
| FAM13A-AS1 | NR_002806 | 0.001694 | 0.393603 |
| FAM172BP | NR_036433 | 0.009426 | 0.492668 |
| AQP7P1 | NR_002817 | 0.007676 | 0.499168 |
| FASN | NM_004104 | 0.005369 | 0.435019 |
| LHX1 | NM_005568 | 0.009504 | 0.452122 |
| FLRT3 | NM_198391 | 0.001269 | 0.4014 |
| CBX2 | NM_005189 | 0.009098 | 0.446245 |
| LOC284561 | XR_110828 | 0.002989 | 0.427585 |

Table S4. List of downregulated genes after *A. actinomycetemcomitans* biofilm challenge with corresponding fold change and p-value. Biofilm group compared to the control group.

| GeneName | SystematicName | p-value | Fold change |
| --- | --- | --- | --- |
| FRY | NM_023037 | 0.007192 | 2.701358 |
| CXCL11 | NM_005409 | 0.008706 | 2.176004 |
| SLC39A2 | NM_014579 | 0.004594 | 2.420143 |
| UPK1A-AS1 | NR_046420 | 0.002396 | 2.20592 |
| CKMT2 | NM_001825 | 0.006759 | 2.383423 |
| PKIB | NM_181795 | 0.005897 | 2.264245 |
| IL2RG | NM_000206 | 0.007678 | 2.29303 |
| CEACAM5 | NM_004363 | 0.005306 | 3.328962 |
| BPIFB1 | NM_033197 | 0.000739 | 4.12133 |
| ZNF160 | BC000807 | 0.007654 | 2.661745 |
| RBM33 | NM_053043 | 0.003428 | 2.109447 |
| ZFYVE9 | ENST00000361625 | 0.002629 | 2.907905 |
| MFSD4 | NM_181644 | 0.009709 | 2.001963 |
| KIAA1244 | NM_020340 | 0.009014 | 2.278109 |
| HM13 | NM_178582 | 0.004082 | 2.157757 |
| CIART | NM_144697 | 0.009994 | 2.266948 |
| PER2 | NM_022817 | 0.0077 | 2.38819 |
| RBM33 | ENST00000287912 | 0.001905 | 2.722232 |
| DNMT3A | NM_175630 | 0.008571 | 2.576692 |
| TUB | NM_003320 | 0.009647 | 2.393906 |
| MDM2 | NM_002392 | 0.009671 | 3.571283 |
| MAB21L1 | NM_005584 | 0.008809 | 2.223689 |
| RNMT | NM_003799 | 0.005942 | 2.569602 |
| BMP3 | NM_001201 | 0.000145 | 2.107785 |
| FRRS1 | NM_001013660 | 0.005383 | 2.391084 |
| TNS1 | NM_022648 | 0.005393 | 7.200253 |
| SVIP | NM_148893 | 0.000544 | 2.023201 |
| BLZF1 | NM_003666 | 0.008894 | 4.035242 |
| MYO1D | NM_015194 | 0.005902 | 2.077204 |
| CEP250 | NM_007186 | 0.008612 | 2.079498 |
| UBE3C | NM_014671 | 0.003748 | 2.07944 |
| DIDO1 | NM_022105 | 0.002432 | 2.229152 |
| KRTAP6-3 | NM_181605 | 0.004761 | 3.455238 |
| FNDC3A | AK056071 | 0.005301 | 4.23286 |
| PTGIS | NM_000961 | 0.001366 | 2.234346 |
| TLR4 | NM_138554 | 0.003631 | 2.068511 |
| AFF4 | NM_014423 | 0.001184 | 2.994754 |
| WDFY3 | ENST00000426414 | 0.006183 | 2.156927 |
| LOC100132356 | NR_034127 | 0.006663 | 2.757469 |
| ERBB3 | NM_001005915 | 0.003444 | 2.86964 |
| F2R | BC016059 | 0.007628 | 2.424286 |
| MPZL3 | NM_198275 | 0.00762 | 4.052789 |
| KDM5C | NM_001146702 | 0.000102 | 2.459193 |
| HIPK2 | NM_001113239 | 0.002113 | 3.253889 |
| FLNA | AK125630 | 0.006975 | 2.39353 |
| LATS1 | NM_001270519 | 0.001978 | 3.194394 |
| KDM4C | BC053678 | 0.007249 | 3.429094 |
| A_33_P3240652 | N/A | 0.008199 | 2.098401 |
| LOC100129675 | AK125061 | 0.008535 | 2.119178 |
| BCL2L11 | NM_207002 | 0.004189 | 2.555982 |
| CEACAM7 | NM_006890 | 0.004022 | 2.742388 |
| MXI1 | NM_005962 | 0.008666 | 2.6549 |
| CBX6 | ENST00000407418 | 0.002325 | 2.618622 |
| A_33_P3273767 | N/A | 0.003056 | 2.721968 |
| LOC101929095 | NR_125911 | 0.001422 | 2.600772 |
| ENST00000495527 | ENST00000495527 | 0.0059 | 2.018733 |
| IKZF2 | NM_001079526 | 0.00178 | 2.122328 |
| ENST00000568091 | ENST00000568091 | 0.006743 | 2.358536 |
| MSANTD3 | NM_001198807 | 0.006948 | 3.278185 |
| AKAP9 | BC015533 | 0.000498 | 3.267121 |
| DCAF5 | NM_001284208 | 0.000865 | 3.503701 |
| TAF1B | ENST00000404869 | 0.006895 | 2.929387 |
| PLEKHM1 | NM_014798 | 0.006681 | 2.277518 |
| THC2719035 | THC2719035 | 0.009504 | 2.539439 |
| ALS2 | ENST00000410052 | 0.009762 | 2.846458 |
| P2RY1 | NM_002563 | 0.004552 | 3.069445 |
| E2F3 | NM_001949 | 0.00522 | 3.722928 |
| ABCA1 | NM_005502 | 0.004648 | 2.259389 |
| ZNF460 | NM_006635 | 0.005067 | 2.687528 |
